# Antiretroviral therapy under the wing of the COVID-19 epidemic: One look, and different solutions

**DOI:** 10.4102/sajhivmed.v21i1.1167

**Published:** 2020-12-15

**Authors:** Heba Elsayed, Mohamed Hassany

**Affiliations:** 1 National AIDS Program, Ministry of Health and Population, Cairo, Egypt; 2 Tropical Medicine Department, National Hepatology and Tropical Medicine Research Institute, Cairo, Egypt; 3 Ministry of Health and Population, Cairo, Egypt

## Introduction

Dear editors, we read with great interest the editorial ‘Antiretroviral therapy optimization in the time of COVID-19: Is it really different in North and South Africa?’ by Cordie et al.^1^ and we find it an excellent opportunity to address some critical issues.

With the detection of the first case of the human immunodeficiency virus (HIV) in Egypt in 1986, the Ministry of Health and Population (MOHP) established the National AIDS Program (NAP) as the entity responsible for combating the HIV epidemic in the country. This task has included prevention and the care and treatment of people living with HIV (PLHIV) and coordinating the efforts of stakeholders such as the United Nations (UN) and civil society organizations (CSOs).^2^

## Antiretroviral therapy procurement strategies in Egypt

Antiretroviral (ARV) drugs were first introduced in Egypt in 2008. From 2008 to 2014, these were funded from the Global Fund to Fight AIDS, tuberculosis and malaria (GFATM). In 2014, the MOHP took the step to start covering ARV drugs from domestic funding and succeeded from 2017 until now to cover 100% of Egypt’s needs from the domestic funds. This has been with a view to ensure ARV-sustainability and avoid the risk of stockouts. This is considered a unique initiative amongst all countries in the region.^3,4^ Egypt has given attention to the procurement and supply management of ART. Clinic pharmacists were provided with a comprehensive medication policy and capacity-building standards through international experts. The latter were recruited by UNICEF, the agency that assists the government in the procurement of unregistered ARVs. This provides ART clinics with a unique stock management system.^5^ In 2018, the MOHP encouraged local pharmaceutical companies to register all first-line ARVs. This covers approximately 97% of the drugs needed by PLHIV. The remaining unregistered ARVs are procured via UNICEF.^5^

## National treatment policies and recommendations

National AIDS Program has expanded the treatment options for PLHIV in accordance with the World Health Organization’s (WHO’s) guidelines and the national context. The most recent Egyptian National Care and ARVs Guidelines were published in 2015. These were followed in 2017 by a summary update. This adopted a ‘Test and Treat/Treat All’ approach that supplies ARVs to all PLHIV regardless of their CD4 count or viral load level. This update included the introduction of dolutegravir (DTG) as an alternative first-line option to efavirenz (EFV) as per the WHO recommendations.^6^

Tenofovir disoproxil fumarate + emtricitabine (TDF + FTC) is the preferred nucleoside reverse-transcriptase inhibitor (NRTI) first-line ART backbone. As per our National guidelines, TDF + FTC + EFV is the currently preferred first-line regimen. An alternative NRTI backbone is the combination of zidovudine (AZT) and 3TC, which is registered and manufactured locally.^7^

## The global effect of coronavirus disease on medication supply chain

With the evolution of the coronavirus disease 2019 (COVID-19) pandemic, all countries have taken measures to control the spread of the infection. As a result of the lockdown and the interference with international flights, global health systems have faced the potential of medicine stock-outs. Seventy-three countries reported such a risk of ARV stockouts.^8^ Based on a recent UNAIDS study that explores challenges facing global HIV programmes, reduced production and availability of active pharmaceutical ingredients (APIs) and issues with transportation have negatively affected dependent regions.^9^ Remedial action has been required by countries and stakeholders.

## National response during coronavirus disease 2019

The Egyptian NAP has taken steps to support PLHIV during the COVID-19 epidemic. These include prolonging ARV-dispensing intervals beyond a month, strengthening teleconsultation services, ensuring ongoing follow-up of COVID-19-infected PLHIV and assisting with their admission to isolation hospitals and subsequent care. Since the start of the pandemic and the confirmation of Egypt’s first COVID-19 case in February 2020,^10^ a ‘partial-lockdown’ model has allowed continued access to medication via a medicines stockpile, the government’s cargo fleet and airline support. These measures protect Egypt from exposure to a significant stockout or delayed ARV access.

## Africa between the hammer of HIV and the anvil of coronavirus disease 2019

Africa has a large HIV burden: ongoing new HIV infections, approximately 500 000 HIV-related deaths in 2018 and at least a third of PLWH still not accessing ART.^11^ Multiple factors have undermined the continent’s ability to end the HIV epidemic. Some of these factors are persisting civil wars, tribal conflicts, natural disasters, poor health systems and weak infrastructure.^12^ These factors have created an ART coverage-gap of 59% in Eastern and Southern Africa, 79% in Western and Central Africa and 89% in North Africa.^13^ This situation has worsened subsequent to the COVID-19 epidemic. Nonetheless a number of North African countries have low HIV prevalence rates, namely less than 0.1% and have been ‘protected’ from these COVID-19-related ART problems.^14^ At the end of September 2020, the number of COVID-19 cases in Africa has approached a million and a half with nearly 35 000 deaths.^15^ A WHO modelling study projects that the number of COVID-19 cases in the first year of the pandemic in Africa will reach between 29 and 44 million and of this between 190 000 and 290 000 will die.^16^ These data suggest a continuous reappraisal of the effects of COVID-19 by African government and the possible ART shortages.

### Key challenges (Figure 1)

Overlapping challenges that may affect the ARVs supply chain:

- Countries’ lockdown measures
- Economic challenges because of the reduction or stoppage of all the economic activities
- The subsequent decrease in API and intermediate pharmaceutical ingredients’ production capacities lead to increased API costs
- Slow shipments and reduced access to medicines.

**FIGURE 1:**
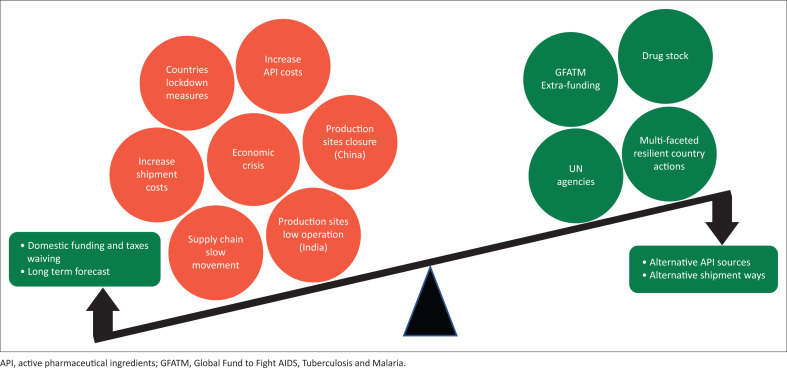
Challenges and solutions in the medication supply chain under coronavirus disease 2019 pandemic.

### Key solutions

With the easing of the lockdown measures the following solutions are surfacing:

- Alternative procurement sources and long-term forecasting plans to enhance drug stocking
- Manipulating different ways of shipping
- Resource mobilisation and the waiving of national taxes to maintain stable pricing levels
- Support from global funding agencies and concerned UN agencies.

## The African Medicine Agency Treaty

The establishment of the African Medicine Agency (AMA) Treaty may assist countries in Africa cope with COVID-19 and HIV.^17^ This coincides with activities of the third Specialised Technical Committee on Health, Population and Drug Control (STC-HPDC-3) held in Cairo in August 2019. Sixteen African countries (Algeria, Benin, Chad, Ghana, Madagascar, Mali, Morocco, Rwanda, Saharawi Arab Democratic Republic, Senegal, Tunisia, Seychelles, Niger, Guinea, Sierra Leone and Burkina Faso) officially signed the treaty. The AMA is considered to be the first continental initiative to integrate the regulations of medicines and medical products thereby ensuring the provision of safe and effective drugs and medical products to the people. The treaty also aims to strengthen Africa’s capacity to produce medicines by utilising available production capacity and unifying standards and product registration across the continent. Such a treaty will allow drugs manufactured or registered in one African country to be deployed elsewhere on the continent without further regulatory constraints. This will in turn widen the availability and variety of drugs and augment competition amongst API providers to ensure that medicines can be offered at an optimum and affordable price.

## Conclusion

The COVID-19 pandemic requires both a short- and long-term strategy to overcome the anticipated shortages of HIV-related healthcare services at national level. In parallel with this, multisectoral international solidarity and collaboration with global stakeholders needs to be continued and fostered to achieve the 90-90-90 UNAIDS HIV-elimination goals and manage COVID-19 successfully.
